# Suitable fertilization can improve maize growth and nutrient utilization in ridge-furrow rainfall harvesting cropland in semiarid area

**DOI:** 10.3389/fpls.2023.1198366

**Published:** 2023-06-08

**Authors:** Jiayi Wang, Gaoxiang Liu, Nan Cui, Enke Liu, Yan Zhang, Donghua Liu, Xiaolong Ren, Zhikuan Jia, Peng Zhang

**Affiliations:** ^1^ College of Agronomy, Northwest A&F University, Yangling, Shaanxi, China; ^2^ Key Laboratory of Crop Physi-Ecology and Tillage Science in Northwestern Loess Plateau, Minister of Agriculture, Northwest A&F University, Yangling, Shaanxi, China; ^3^ Institute of Environment and Sustainable Development in Agriculture, Chinese Academy of Agricultural Sciences, Beijing, China; ^4^ Institute of Jiangxi Oil-tea Camellia, Jiujiang University, Jiujiang, Jiangxi, China

**Keywords:** fertilizer rate, ridge-furrow rainfall harvesting system, maize yield, nutrient uptake and utilization, semi-arid area

## Abstract

The ridge-furrow rainfall harvesting system (RFRH) improved the water shortages, and reasonable fertilization can promote nutrient uptake and utilization of crops, leading to better yield in semi-arid regions. This holds significant practical significance for improving fertilization strategies and reducing the application of chemical fertilizers in semi-arid areas. This field study was conducted to investigate the effects of different fertilization rates on maize growth, fertilizer use efficiency, and grain yield under the ridge-furrow rainfall harvesting system during 2013-2016 in semiarid region of China. Therefore, a four-year localization field experiment was conducted with four fertilizer treatments: RN (N 0 kg hm^-2^, P_2_O_5_ 0 kg hm^-2^), RL (N 150 kg hm^-2^, P_2_O_5_ 75 kg hm^-2^), RM (N 300 kg hm^-2^, P_2_O_5_ 150 kg hm^-2^), and RH (N 450 kg hm^-2^, P_2_O_5_ 225 kg hm^-2^). The results showed that the total dry matter accumulation of maize increased with the fertilizer application rate. The nitrogen accumulation was highest under the RM treatment after harvest, average increase by 1.41% and 22.02% (P<0.05) compared to the RH and RL, respectively, whereas the phosphorus accumulation was increased with the fertilizer application rate. The nitrogen and phosphorus use efficiency both decreased gradually with the fertilization rate increased, where the maximum efficiency was observed under the RL. With the increase of fertilizer application rate, the maize grain yield initially increased and then decreased. Under linear fitting, the grain yield, biomass yield, hundred-kernel weight, and ear-grain number all showed a parabolic trend with the increase of fertilization rate. Based on comprehensive consideration, the recommended moderate fertilization rate (N 300 kg hm^-2^, P_2_O_5_ 150 kg hm^-2^) is suitable for the ridge furrow rainfall harvesting system in semiarid region, and the fertilization rate can be appropriately reduced according to the rainfall.

## 1 Introduction

Drought and infertile soils are the major constraints to maize production in semiarid areas in China (Jia et al., 2020). Limited irrigation water and unstable rainfall in arid agriculture make it difficult to increase nutrient uptake, which may pose a threat to maize production in this regions (Gan et al., 2013; Wu and Ma, 2015). Over the past 20 years, the ridge-furrow rainfall harvesting planting (RFRH) system has been an effectively solution to alleviate issues of drought and water scarcity in dryland areas by reducing evaporation, conserving soil moisture, and increasing the utilization rate of precipitation to over 65%, and also resulted in nearly quadruple grain yields (Ren et al., 2017; Zhang et al., 2018). Fertilization is one of the primary method for improving soil infertile, as it enhances the soil water retention capacity and utilization efficiency, leading to improved photosynthesis and nutrient absorption and utilization by crops (Zhang et al., 2017). However, long-term excessive application of the chemical fertilizer has caused many negative effects, such as plant growth stunted, nutrient leaching, and declining soil quality, which can subsequently limit crop yields in these areas (Lian et al., 2016). Thus, it is necessary to scientifically determine the appropriate application rates of N and P fertilizers under RFRH system, to achieve the purpose of reducing fertilizer usage while increasing efficiency, and maintain the sustainable development In semiarid region.

The plastic film mulching is also beneficial for the dissolution and diffusion of soil nutrients, significantly increasing the concentration of nitrogen, phosphorus, potassium and organic matter in the soil, thus promoting the better efficient utilization of nutrients, growth and production of crops (Wang et al., 2022). Li et al. (2018) demonstrated that in the semi-arid western region of northeast China, application of fertilizer ranging from 70 to 210 kg hm^-2^ can maintain high level of yield, nutrient absorption and utilization, while the dry matter accumulation and yield are both negatively impacted with the increase of fertilizer application. In the arid region of northwest China, applying N at a rate of 265.0kg hm^-2^ and P_2_O_5_ at a rate of 132.5 kg hm^-2^ can maintain the yield and nutrient absorption and utilization at a high level, while the dry matter accumulation and yield are both affected with the continuous increase of fertilizer application.(Zhang et al., 2018). Ren et al. (2010) found that the RFRH system can promote the effectiveness of soil moisture for plant roots and enhance crop nutrient uptake. Xu et al. (2020) found that N 140 kg hm^-2^ for maize and N 240 kg hm^-2^ for wheat in the rotation of maize and winter wheat could achieve high production benefits in the North China Plain. In addition, Lian et al. (2016) also found that the application of N and P fertilizers at a ratio of 186:96 kg N:P hm^-1^ in RFRH system can significantly increase the height of foxtail millet, as well as the chlorophyll content and leaf area index, which can provide a foundation for the accumulation of dry matter and increased yield.

Previous studies on the RFRH system have focused mainly on water use efficiency, ridge-to-furrow ratio, irrigation, and covering materials (Ren et al., 2016a; Ren et al., 2016b; Zhang et al., 2019). However, there is still a lack of systematic evaluation of the effects of different rainfall patterns combined with N:P fertilization levels on nutrient accumulation, distribution, utilization, and yield. We hypothesized that: (1) As The maize yield could not increase with the fertilizer application rate increases under the RFRH system; (2) Different precipitation and fertilization would significantly affect the yield and nutrient uptake and utilization; (3) There would be differences in the effects of water-fertilizer relationship on yield an nutrient utilization. Thus, we conducted a four consecutive years of field positioning experiments with the aim of providing a theoretical basis and technical support for increasing yield and efficiency, and rational fertilizer management of dryland maize cultivation, and further tapping the potential for increasing production in arid areas. The objectives of our study were as follows: (1) Investigated the effects of different fertilizer application rates on crop growth; (2) Compared the improve effects on maize nutrient uptake and utilization with different fertilizer application rates; (3) Analyze the relationship between different water-fertilizer relationships and the yield as well as its components, develop a suitable fertilizer application model for the local area.

## 2 Materials and methods

### 2.1 Site description

**Figure 1:**
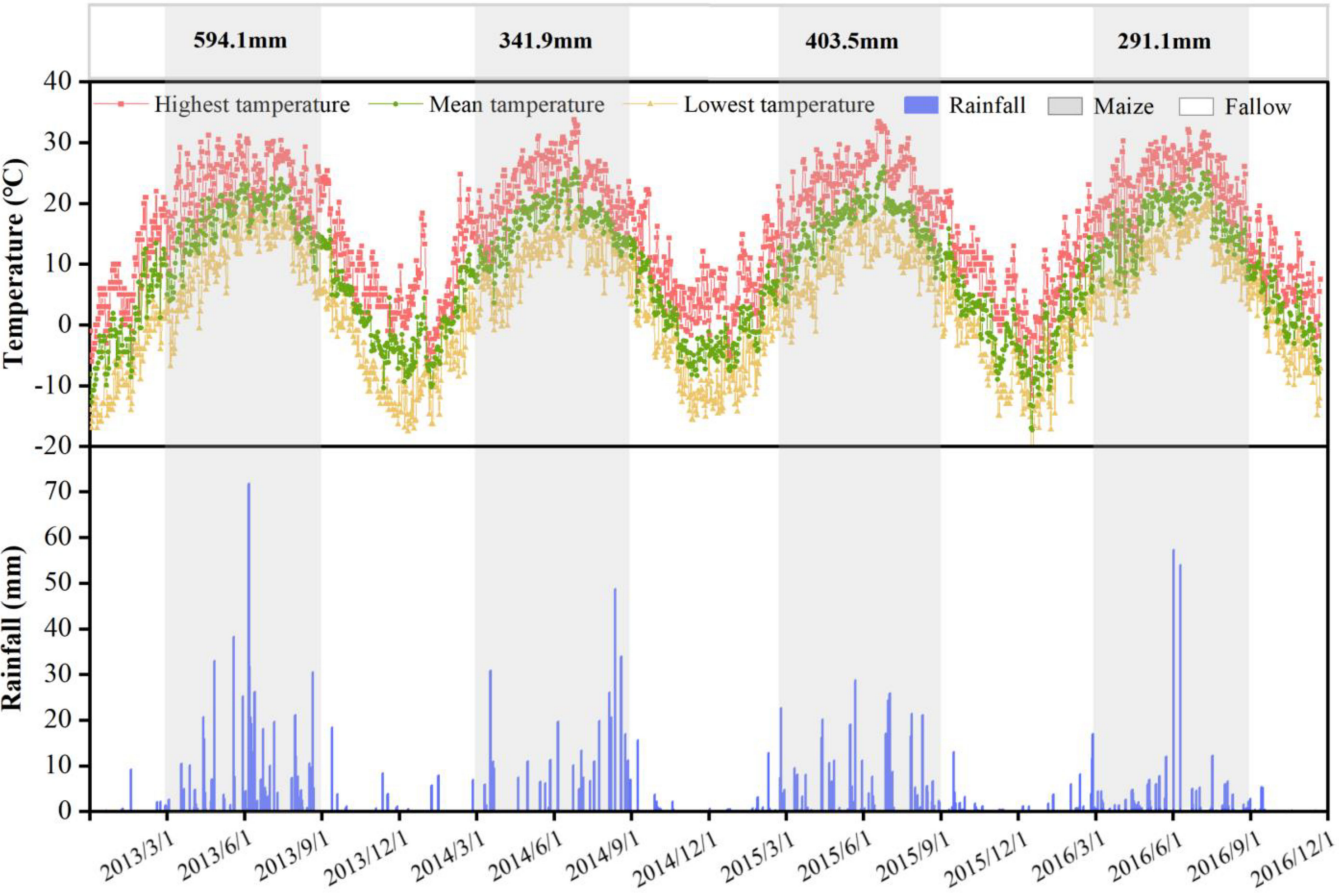
The daily precipitation, maximum, minimum, and average temperatures during the experimental period from 2013 to 2016.

The study was conducted during 2013-2016 at the Dry-land Agricultural Experiment Station of Northwest A&F University in Pengyang, Guyuan, Ningxia, China (35°51’ E, 106°48’ N), which is located in the Loess Plateau region, with a temperate semi-arid climate zone and an elevation of 1658 meters. The annual mean precipitation was 430 mm yr^-1^(according to **Figure 1**), the annual mean temperature was 6.1°C, the total sunshine hours duration 2518.2 h yr^-1^, the annual evaporation was 1753.2 mm, and the frost-free season was 140-160 days. The soil at the experimental site was loess soil, and the main physico-chemical properties of the plow layer (0-60 cm) are as follows: organic matter = g kg^-1^, available nitrogen = 51.75 mg kg^-1^, available phosphorus = 17.17 mg kg^-1^, available potassium = 7.02 mg kg^-1^, total nitrogen = 1.06 g kg^-1^, soil pH = 8.5, and bulk density = 1.25 g cm^-3^.

### 2.2 Experimental design and field management

**Figure 2:**
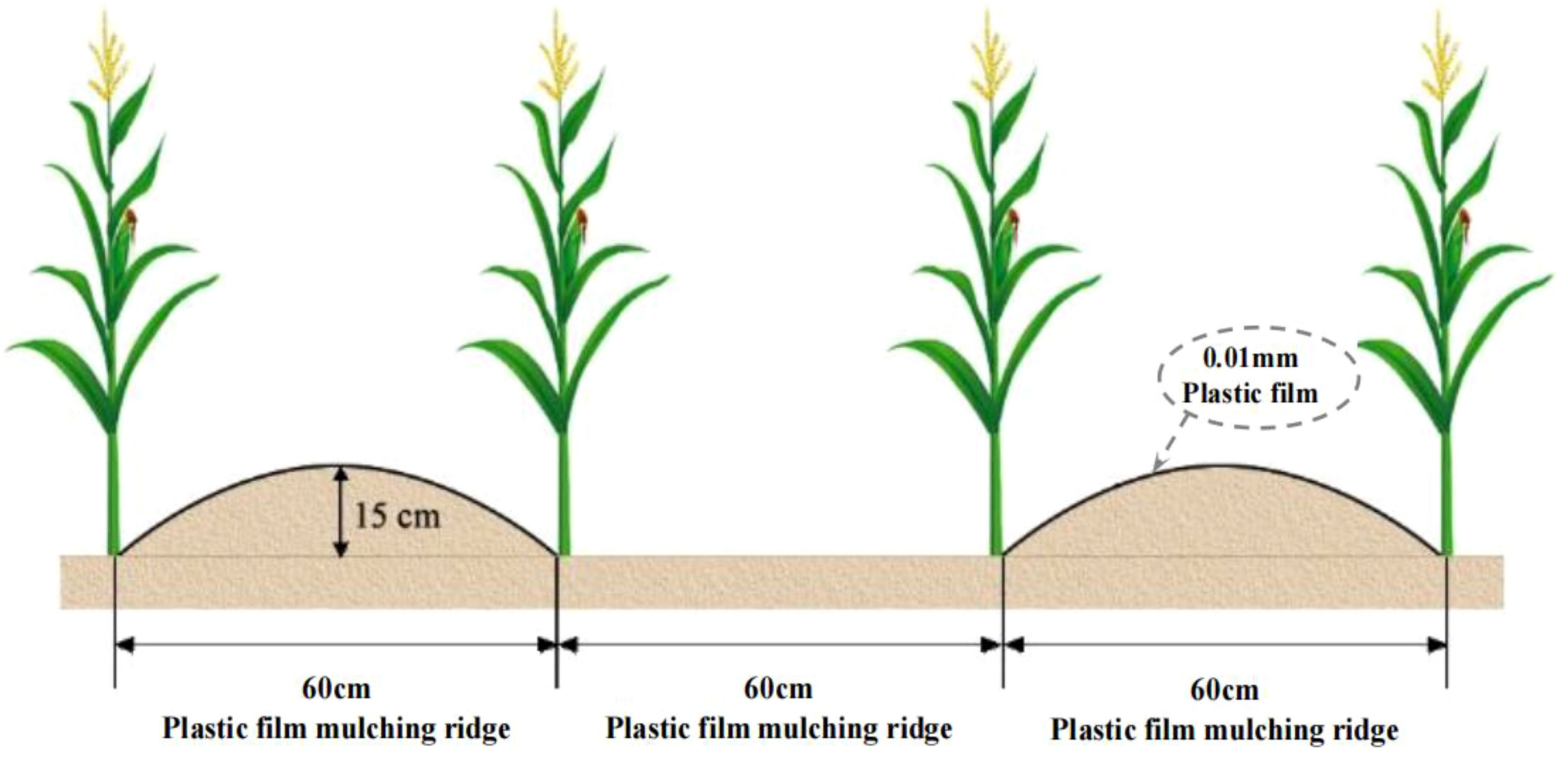
The ridge-furrow rainfall harvesting planting pattern diagram.

The study was conducted using a completely randomized block design with three replicates and for a total of 12 plots. The field studies consisted of four fertilization treatments implemented in the ridge-furrow rainfall harvesting planting system (**Figure 2**): RFRH system with no fertilizer (RN, N 0 kg hm^-2^, P_2_O_5_ 0 kg hm^-2^), RFRH system with low fertilizer (RL, N 150 kg hm^-2^, P_2_O_5_ 75 kg hm^-2^), RFRH system with medium fertilizer (RM, N 300 kg hm^-2^, P_2_O_5_ 150 kg hm^-2^), and RFRH system with high fertilizer (RH, N 450 kg hm^-2^, P_2_O_5_ 225 kg hm^-2^). The plots were 90 cm apart, and each plot area measured 61.2 m^2^ (17 × 3.6 m). The ridges and furrows were both 60 cm wide, with a ridge height of 15 cm, and the ridges were covered with polyethylene plastic film mulch (75 cm wide, 0.01 mm thick, produced by Gansu Tianbao Plastic Industry Co., Ltd.).

Maize (Dafeng 30) was planted at a rate of 75,000 plants hm^-2^. The seeds were sown in mid to late April using a manual falcon beak planter. The maize was manually harvested in late September or early October and all straw was removed from the field. The plastic film was kept in field until 30 days before sowing, at which point it was removed, and the field was prepared for the next planting. Urea (N≥46%) and ammonium phosphate (P_2_O_5_≥46%) were used as fertilizers. Before sowing, uniformly applied all the phosphorus and 60% of the nitrogen as basal fertilizer to the plot. The other 40% of the nitrogen was applied during the early tasseling period of the maize (65-75 days after sowing). Throughout the maize growth period, no irrigation was applied, and weed control and pesticide application were carried out as commonly practiced by local farmers.

### 2.3 Sampling and measurements

#### 2.3.1 Plant height and leaf area plant^-1^

Five randomly selected plants were sampled from each plot to measure plant height and leaf area after 30, 60, 85, 105, and 129 days following maize sowing. The leaf area was calculated using Equation (1):


(1) $$S=\sum_{1}^{i}L\times W\times 0.75$$

where *S* is the total area (cm^2^) of a fully expanded leaf, *L* is the leaf length (cm), *W* is the leaf maximum width (cm), *0.75* is the conversion coefficient, and *i* is the number of leaves.

#### 2.3.2 Dry matter

Three randomly plants were selected from each plot at 30, 60, 85, 105, 129 and 170 days after maize sowing to measure the dry matter accumulation. The maize plants and different plant sections were weighed separately after oven dried at 105°C for 30 min, and then dried at 65°C until a constant weight was reached.

#### 2.3.3 Yield and yield components

At harvest, three randomly rows of maize plants were selected from each plot to measure yield-related traits (ear grains and 100-kernel weight). After threshing and sun drying until the moisture content was less than 14%, then weighed to calculate the grain yield.

#### 2.3.4 Plant nutrient

After the maturity stage of maize, three randomly plants were selected from each plot. Leaves, stalks, grains, husks, and the cob of each maize plant were collected, oven dried at 105°C for 30 minutes, and then dried at 65°C to a constant weight. Samples were finely ground and digested using H_2_SO_4_-H_2_O_2_. The N content in plant samples was analyzed using the Kjeldahl method, and the P content was determined using the molybdenum antimony anti-colorimetric method.

The NUE and PUE were calculated using Equation (2) and (3), respectively (Duan et al., 2014; Langholtz et al., 2021):


(2) $$NUE=100\%\times (N_{F}-N_{0})/F_{N}$$

(3) $$PUE=100\%\times (P_{F}-P_{0})/F_{P}$$

where NUE and PUE are the N and P use efficiency (kg kg^-1^), N_F_ and P_F_ are the N and P uptake amounts (kg hm^-2^) in the N and P fertilization treatments, N_0_ and P_0_ are the N and P uptake amounts (kg hm^-2^) in the no N and P fertilization treatments, F_N_ and P_N_ are the N and P application rates (kg hm^-2^).

The NPE and PPE were calculated using Equation (4) and (5), respectively (Rochester, 2011):


(4) $$NPE=(GY_{NF}-GY_{N0})/(N_{F}-N_{0})$$

(5) $$PPE=(GY_{PF}-GY_{P0})/(P_{F}-P_{0})$$

where NPE and PPE are N and P uptake efficiency (kg kg^-1^), GY_NF_ and GY_PF_ are the grain yield under N and P fertilization treatments (kg hm^-2^), GY_N0_ and GY_P0_ are the grain yield under no N and P fertilization treatments (kg hm^-2^), N_F_ and P_F_ are the N and P uptake under N and P fertilization treatments (kg hm^-2^), and N_0_ and P_0_ are the N and P uptake under no N and P fertilization treatments (kg hm^-2^).

The NHI and PHI were calculated using Equation (6) and (7), respectively:


(6) $$NHI=N_{GY}/N_{BY}$$

(7) $$PHI=P_{GY}/P_{BY}$$

Where NHI and PHI are N and P harvest index (%), N_GY_ and P_GY_ are N and P uptake in the grains under N and P fertilization treatments (kg hm^-2^), and N_BY_ and P_BY_ are the N and P uptake in the above-ground parts under no N and P fertilization treatments (kg hm^-2^), respectively.

### 2.4 Data analysis

The experimental data were processed using Microsoft Excel. Single-factor analysis of variance was performed using SPSS Statistics 25, and multiple comparisons between different treatments were conducted using the Duncan’s new multiple range test at a significance level of (P< 0.05). Origin 2022 was used for graphing.

## 3 Results

### 3.1 Growth of maize under different fertilizer rates

#### 3.1.1 Plant height

**Figure 3:**
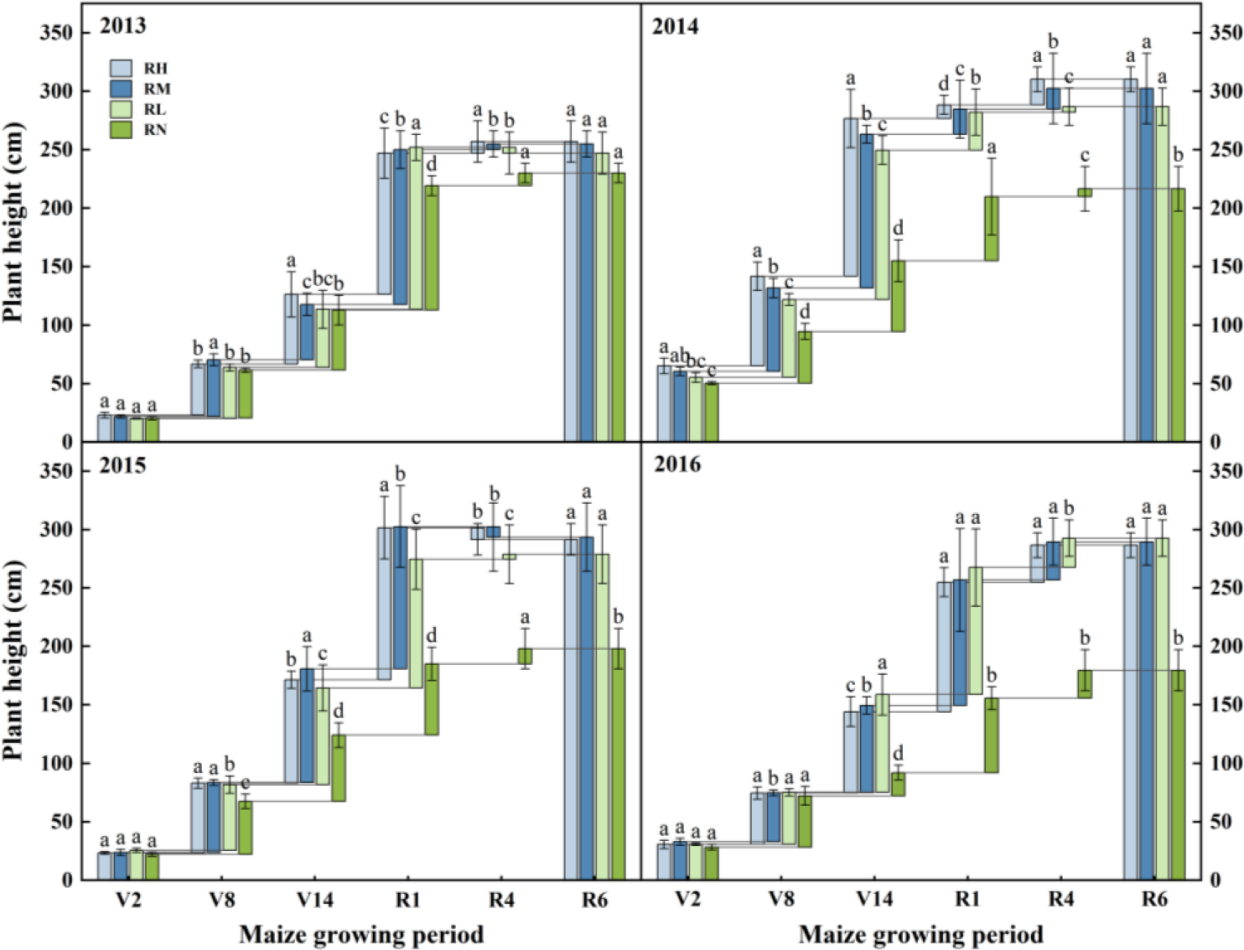
Growth rates of maize plant height under different fertilization levels during various growth periods from 2013 to 2016. Treatments RH, RM, RL, and RN represent applied N:P_2_O_5_ rates of 0:0, 150:75, 300:150, and 450:225 kg hm^–1^, respectively. Data are means ± SD (n=3). Lower case letters indicate significant differences among treatments. Bars represent standard deviations (LSD test, P< 0.05). The period are as follows: V2, second leaf, about 30 days after sowing; V8, eighth leaf, about 60 days after sowing; V14, fourteenth leaf, about 85 days after sowing; R1, silking, about 105 days after sowing; R4, dough, about 129 days after sowing; R6, physiological maturity.

During 2013-2016, the maize height showed an “S” shaped growth trend during the growth period, and reaching maximum in the R4 stage (**Figure 3**). The fertilization treatments were all significantly (*P<0.05*) higher than the unfertilized treatment RN, and the differences gradually increased with the fertilizer application years. Compared with RN, the plant height of RH, RM, and RL average increased by 22.67%, 22.40%, and 21.03%, respectively. In addition, due to the cumulative effects, the difference between the three fertilization treatments was gradually decreased with the fertilizer application years increase. In the early stage of experiment (2013-2014), the order of the fertilization treatments was RH>RM>RL, and the plant height of RH was significantly (*P<0.05*) higher than RM and RL by 16.65% and 20.61% in average in early growth stages (V2 and V8), respectively. In 2016, the order was changed to RL>RM>RH, and the plant height did not differ significantly between the treatments at each growth stages.

#### 3.1.2 Plant leaf area

**Figure 4:**
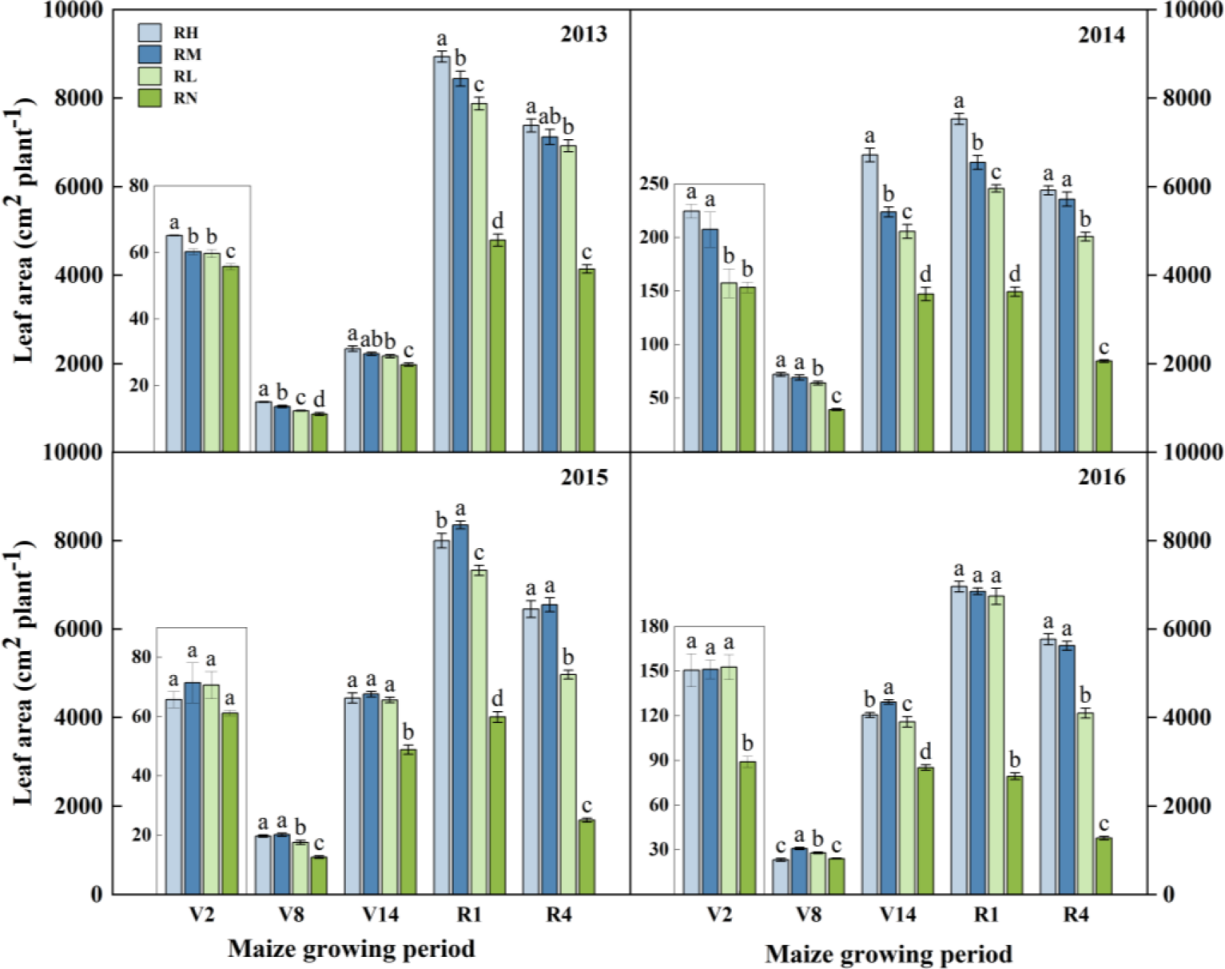
Leaf area of maize under different fertilization levels during various growth periods from 2013 to 2016. Treatments RH, RM, RL, and RN represent applied N:P_2_O_5_ rates of 0:0, 150:75, 300:150, and 450:225 kg hm^–1^, respectively. Data are means ± SD (n=3). Lower case letters indicate significant differences among treatments. Bars represent standard deviations (LSD test, P< 0.05). The period are as follows: V2, second leaf, about 30 days after sowing; V8, eighth leaf, about 60 days after sowing; V14, fourteenth leaf, about 85 days after sowing; R1, silking, about 105 days after sowing; R4, dough, about 129 days after sowing; R6, physiological maturity.

The leaf area of maize plants in response to fertilization showed an “S” shaped (**Figure 4**), and the maximum during the tasseling and flowering stage (R1). The difference between the fertilizer treatments was different with the increase of fertilizer application years. In 2013-2014, the order of leaf area throughout the growth period was RH>RM>RL>RN, the leaf area under RH and RM was higher than RL by 13.58% (*P<0.05*) and 6.45% in average, respectively. In 2015, the order of treatments was RM>RH>RL>RN at all growth stages (except V2), and there were no significant differences between the fertilization treatments during the vegetative period (V2-V14), whereas RH and RM were significantly (*P<0.05*) higher than RL during the R1 and R4 stages, by 15.68% and 18.20%, respectively. In 2016, the differences between fertilization treatments gradually decreased. During the V8 and V14 stages, compared with RH and RL, leaf area significantly (*P<0.05*) increased under RM by 19.99% and 9.91%, respectively, whereas during the R1 and R4 stages, RH and RM were higher than RL, but leaf area did not significant difference between these two treatments.

#### 3.1.3 Dry matter

**Figure 5:**
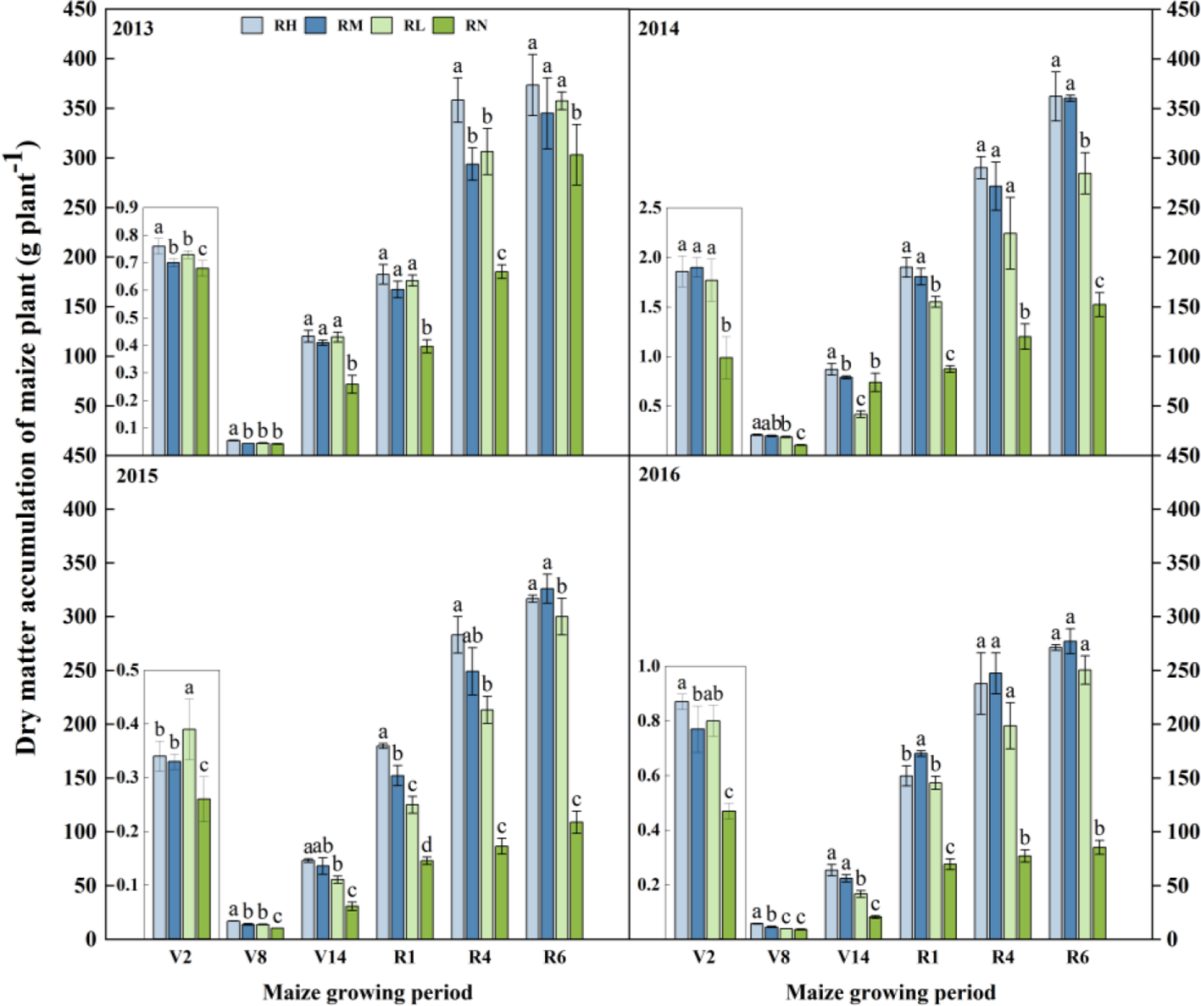
Dry matter of maize under different fertilization levels during various growth periods from 2013 to 2016. Treatments RH, RM, RL, and RN represent applied N:P_2_O_5_ rates of 0:0, 150:75, 300:150, and 450:225 kg hm^–1^, respectively. Data are means ± SD (n=3). Lower case letters indicate significant differences among treatments. Bars represent standard deviations (LSD test, P< 0.05). The period are as follows: V2, second leaf, about 30 days after sowing; V8, eighth leaf, about 60 days after sowing; V14, fourteenth leaf, about 85 days after sowing; R1, silking, about 105 days after sowing; R4, dough, about 129 days after sowing; R6, physiological maturity.

The dry matter accumulation was gradually decreased with the fertilizer application years (**Figure 5**). All fertilization treatments were significantly higher than unfertilized treatment at each growth stages, and the differences was gradually increased with the fertilization years. During 2013-2014, there was little difference among the fertilization treatments, only RH significantly increased by 11.47% (*P<0.05*) compared to RL. In 2015, the fertilization treatments at each growth stage (except V2 stage) followed the order as RH>RM>RL. Compared with RL, the dry matter accumulation under RH and RM average increased by 20.64% (*P<0.05*) and 12.10%, respectively. Due to the cumulative effects of fertilizer application, the dry matter in the late growth stages (R1-R6) decreased by 6.60% under RH than RM, and only increased by 17.12% under RH compared to RL.

#### 3.1.4 Dry matter accumulation of different organs

**Figure 6:**
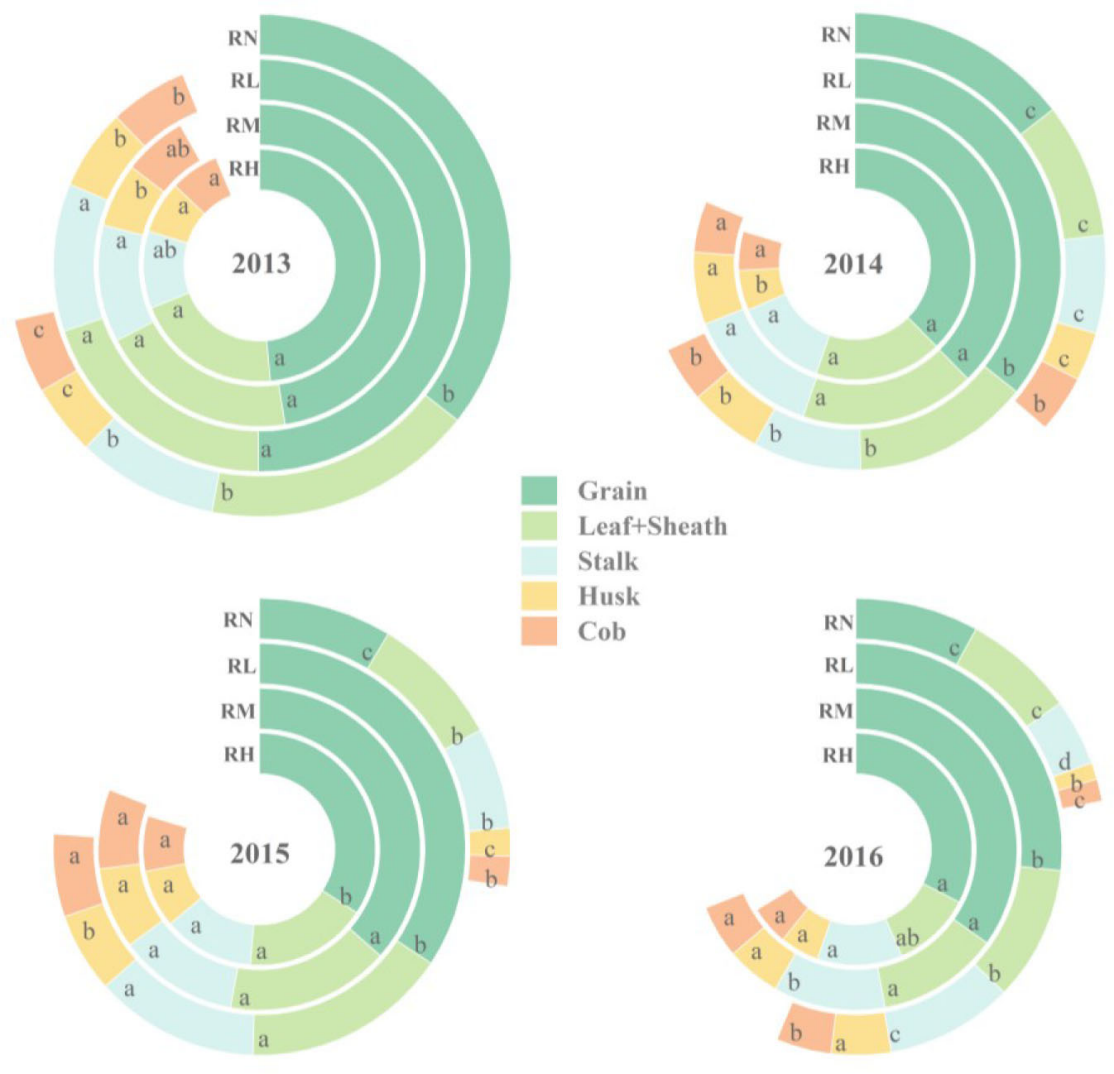
Dry matter accumulation of different organs of maize under different fertilization levels during harvest period from 2013 to 2016. Treatments RH, RM, RL, and RN represent applied N:P_2_O_5_ rates of 0:0, 150:75, 300:150, and 450:225 kg hm^–1^, respectively. Lower case letters indicate significant differences among treatments.

The dry matter distribution of different organs under fertilization treatments was significantly (*P<0.05*) higher than the RN at harvest, and gradually decreased with the fertilization years (**Figure 6**). The dry matter mainly accumulated in the grains, which accounting for an average proportion of 46.13%. In 2014 and 2016, the dry matter of different organs significantly increased by 14.42% and 11.58% under RM and RH compared with RL. In 2015, compared with RH and RL, the average dry matter of different organs significantly (*P<0.05*) increased by 6.26% and 5.89% under the RM, respectively. Moreover, the dry matter accumulation of maize stem was highest in RM during 2013-2014, average increased by 19.62% and 6.66% under RM compared to RL and RH, respectively. In 2016, compared with RM and RL, the dry matter accumulation significantly (*P<0.05*) increased by 5.02% and 15.33% under RH. In addition, the leaves dry matter of RM and RH were 20.92% (*P<0.05*) and 22.08% (*P<0.05*) higher than RL in 2014, respectively. In 2016, the leaves dry matter accumulation of RM was 13.38% (*P<0.05*) higher than RL. Moreover, the dry matter proportion of maize husks and cobs was relatively low, with the similar trends among each experimental year. Compared with RL, the dry matter of husks average increased by 11.49% and 14.68% under RH and RM, and the cobs average increased by 16.70% and 14.14%, respectively.

### 3.2 Accumulation of N and P under different fertilizer rates

#### 3.2.1 N and P accumulation

The total N uptake under fertilization treatments was higher than RN in each experimental year, and it was gradually decreased with the fertilization years (**Table 1**). In 2013, the order of N accumulation under fertilization treatments was RH>RM>RL, and only the accumulation of RH was 10.14% (*P<0.05*) higher than RL. As the years of fertilization increased, the differences among the fertilization treatments were increased. During 2014-2016, the order of total N accumulation changed to RM>RH>RL, and the N accumulation of RM and RH was 22.38% (*P<0.05*) and 19.77% (*P<0.05*) higher than RL, respectively. Moreover, compared with RH, the N accumulation of RM significantly (*P<0.05*) increased by 2.94% and 5.23% in 2014 and 2015, respectively.

**Table 1:** Effects of years and fertilizer rate on accumulation of N and P for maize during harvest period from 2013 to 2016.

Treatment	N accumulation (kg hm^-1^)				P accumulation (kg hm^-1^)			
	2013	2014	2015	2016	2013	2014	2015	2016
RH	399.80a ± 30.31	315.38b ± 4.38	277.63b ± 6.81	291.17a ± 28.68	34.27a ± 2.57	38.07a ± 1.26	22.89a ± 1.00	15.41a ± 1.02
RM	389.81ab ± 15.26	324.92a ± 4.82	292.96a ± 3.46	294.41a ± 5.69	31.40ab ± 0.64	34.19b ± 0.72	19.75b ± 0.75	13.50ab ± 0.30
RL	359.28b ± 13.95	249.99c ± 6.29	249.22c ± 5.01	208.60b ± 20.16	29.32b ± 0.27	26.87c ± 0.27	16.37c ± 0.32	9.12b ± 0.27
RN	252.76c ± 12.92	93.89d ± 1.66	69.64d ± 5.33	45.48c ± 4.95	24.98c ± 1.74	11.15d ± 0.23	3.77d ± 0.99	2.47c ± 0.30
ANOVA								
Year(Y)	***<0.001				***<0.001			
Fertilization(F)	***<0.001				***<0.001			
Y×F	***<0.001				***<0.001			

Treatments RH, RM, RL, and RN represent applied N:P_2_O_5_ rates of 0:0, 150:75, 300:150, and 450:225 kg hm^–1^, respectively. Data are means ± SD (n=3). Lower case letters indicate significant differences among treatments. Bars represent standard deviations (LSD test, P< 0.05). In Analysis of Variance, Y, F and Y×F represents the year, fertilizer and the interaction between the year and fertilizer. *** Significant differences at P < 0.001.

Similar to the N uptake of maize, the P uptake of fertilization treatments was higher than RN (**Table 1**). Among the fertilization treatments, the P accumulation under RH and RM were significantly (*P<0.05*) higher than the RL, with average increased by 28.28% and 19.39% during 2013-2016, respectively. However, in 2014 and 2015, the P accumulation was significantly (*P<0.05*) increased by 10.19% and 13.71% under RH compared to RM, respectively.

#### 3.2.2 N and P accumulation in different organs

**Figure 7:**
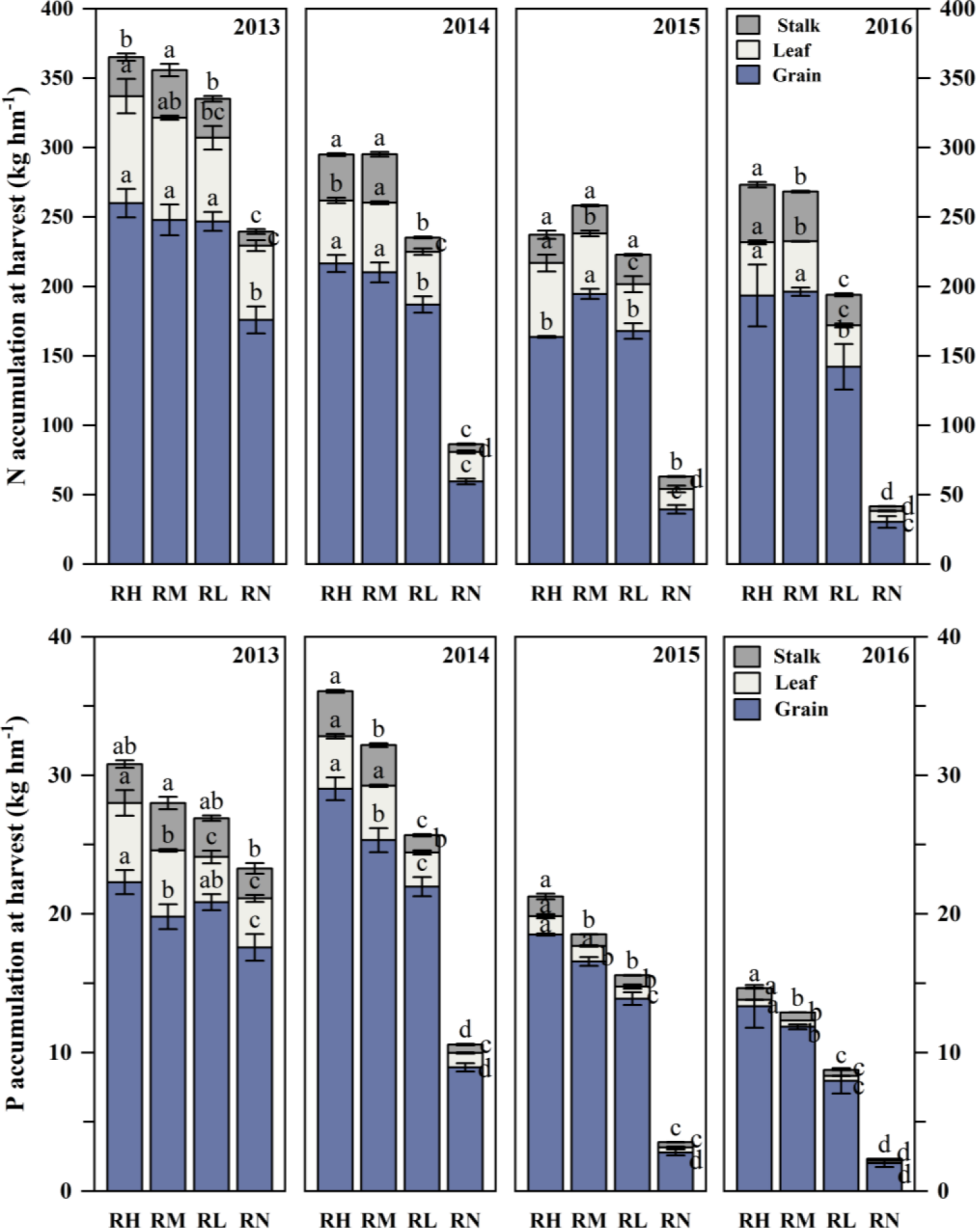
Accumulation of N and P in maize grains, leaves, and stalks under different fertilization levels during harvest period from 2013 to 2016. Treatments RH, RM, RL, and RN represent applied N:P_2_O_5_ rates of 0:0, 150:75, 300:150, and 450:225 kg hm^–1^, respectively. Lower case letters indicate significant differences among treatments. Bars represent standard deviations (LSD test, P< 0.05).

The order of N accumulation in different organs was grain > leaf > stalk > sheath > husk after harvest (**Figure 7A**). The N uptake of each organ under fertilization treatments was higher than RN, which was decreased gradually except for the stem with the fertilization years. In 2013-2014, the order of N accumulation in grain under fertilization treatments was RH > RM > RL > RN, and RH was average higher than RM, RL and RN by 3.82%, 9.38%, and 52.45% (*P<0.05*), respectively. The leaves have the similar trend in each year, the average N accumulation of RH and RM were all significantly higher than RL, average increased by 24.26% and 20.81%, respectively, but RH was 9.25% higher compared with RM in 2014. Furthermore, the stems N accumulation showed large differences among different years. In 2013, the RM significantly (*P< 0.05*) increased by 18.10% and 18.28% compared with RH and RL, respectively. In 2014, the RM and RH were 70.60% (*P< 0.05*) and 68.91% (*P< 0.05*) higher than RL. And no significant differences between the fertilization treatments in 2015. In 2016, the order of stems N accumulation was RH > RM > RL, the RH was 13.72% (*P<0.05*) and 46.99% (*P<0.05*) higher than that under RM and RL, respectively.

The phosphorus accumulation in different organs followed the order as grain > leaf > stalk > sheath > husk (**Figure 7B**). The P uptake under each fertilization treatment was higher than RN, and gradually decreased with the fertilization years. Analysis of P uptake in different organs showed that the grain P accumulation under each fertilization treatment first increased and then decreased with the fertilization years, reaching the maximum value in 2014. But the order changed to RH > RM > RL in 2013, and RH significantly (*P< 0.05*) increased by 11.44% and 29.87% compared to RM and RL, respectively. Besides, the leaf P accumulation under RH and RM in each year was significantly (*P< 0.05*) higher than RLby 34.33% and 28.24% in average, respectively. Except for 2014, RH was slightly higher than RM, but significantly increased (*P< 0.05*) by 16.28% and 3.87% in 2013 and 2016, respectively. Furthermore, stems P accumulation in each experimental year (except for 2013) followed the order of RH > RM > RL, RH significantly (*P< 0.05*) increased by 27.05% and 51.00% compared with RM and RL, respectively, and RM was only 57.83% (*P< 0.05*) and 24.65% (*P< 0.05*) significantly higher compared with RL in 2014 and 2016, respectively.

### 3.3 Yield effects of different fertilizer rates and rainfall

The effects of year and fertilization on grain and biomass yield were extremely significant (*P< 0.001*). The interaction of year and fertilization had an extremely significant effect on ear grains, 100-kernel weight, and grain yield (**Table 2**). As fertilizer application rates increased, the ear grains and grain yield increased followed by a decreasing trend, reaching the maximum value under RM. Compared with the RH and RL, the ear grains increased by an average of 3.62% and 3.79% under RM, and the grain yield was increased by 2.93% and 3.27%, respectively.

**Table 2:** Maize ear grains, 100-kernel weight, grain yield and biomass yield for different fertilization rates from 2013 to 2016, and the Analysis of Variance between years and fertilization rates.

Year	Treatment	Grains per ear	100-kemel weight (g)	Grain yield (Mg hm^-^¹)	Biomass yield (Mg hm^-1^)
2013	RH	606.79a ± 12.65	29.13ab ± 0.80	12.36a ± 0.49	28.0la ± 3.26
	RM	618.9la ± 17.60	27.92b ± 0.49	12.0la ± 0.54	26.87b ± 1.36
	RL	589.63b ± 10.16	30.02a ± 1.28	12.77a ± 0.35	26.81b ± 1.95
	RN	499.18c ± 23.08	25.42c ± 1.25	9.75b ± 0.78	22.74c ± 7.66
2014	RH	486.29a ± 30.36	32.46a ± 0.73	10.79ab ± 0.12	27.17a ± 1.84
	RM	485.86a ± 20.47	31.96a ± 0.45	11.10a ± 0.59	27.03a ± 2.46
	RL	469.57a ± 43.88	29.42b ± 0.82	10.17b ± 0.43	21.36b ± 2.25
	RN	308.79b ± 113.60	21.05c ± 0.39	4.03c+0.39	10.76c ± 0.38
2015	RH	519.98b ± 27.07	27.85a ± 0.64	10.26a ± 0.46	22.75a ± 0.64
	RM	574.45a ± 14.47	27.72a ± 0.63	10.94a ± 0.57	23.45a ± 0.97
	RL	536.00b ± 28.53	27.07a ± 1.06	10.30a ± 1.31	22.17a ± 1.64
	RN	162.08c ± 13.98	21.12b ± 1.79	2.57b ± 0.34	8.17b ± 0.58
2016	RH	550.18a ± 25.20	23.36a ± 0.12	8.92a ± 0.55	20.35a ± 1.01
	RM	561.70a ± 8.41	23.15ab ± 0.25	9.37a ± 0.29	20.79a ± 0.40
	RL	559.55a ± 9.51	22.74ab ± 0.34	8.88a ± 0.23	18.78b ± 1.84
	RN	135.65b ± 3 29	22.12b ± 1 32	1.83b ± 0 36	6.43c ± 0 64
Analysis of Variance					
Year (Y)		**	*	***	***
Fertilization (F)		**	*	***	***
Y×F		***	***	***	**

Treatments RH, RM, RL, and RN represent applied N:P_2_O_5_ rates of 0:0, 150:75, 300:150, and 450:225 kg hm^–1^, respectively. Lowercase letters indicate significant differences among treatments (LSD test, P<0.05). Data are means ± SD (n=3). Mean values within a column in each year followed by the same letter are not significantly different at p< 0.05. In Analysis of Variance, Y, F and Y×F represents the year, fertilizer and the interaction between the year and fertilizer. *** Significant differences at P < 0.001; ** significant differences at P < 0.01; * significant differences at P < 0.05.

All fertilization treatments significantly (*P<0.05*) increased the ear grain number by an average increase of 49.75% in each year. Also, differences in fertilization effects and rainfall produced differences among fertilization treatments. In 2013, the average ear grains significantly (*P<0.05*) increased under RH and RM by 2.83% and 4.73%, respectively. In 2015 and 2016, the order of fertilization treatments was RM>RL>RH with the fertilization years, and the RM and RL were 6.29% (*P<0.05*) and 2.39% higher compared with RH, respectively.

The 100-kernel weight was significantly affected by fertilization, which increased with the fertilizer application rate in the early stage, but the differences diminished gradually with the years of fertilization. Except for 2013, the order of three treatments was RH>RM>RL. In 2014, the average 100-kernel weight was 9.37% (*P<0.05*) and 7.95% (*P<0.05*) higher under RH and RM compared with RL, respectively, but there were no significant differences among the treatments in other years.

**Figure 8:**
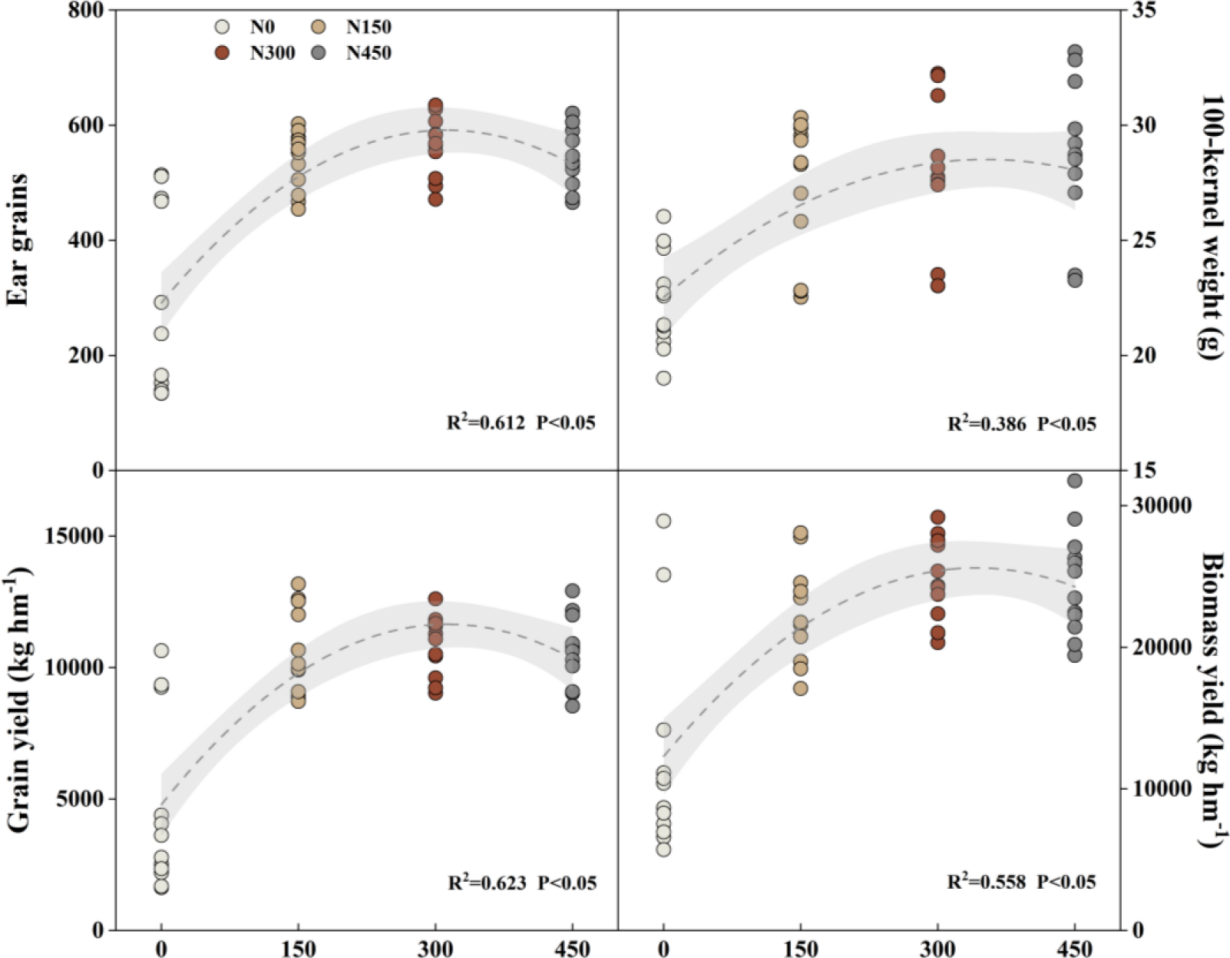
Linear regression relationships between different fertilization levels and maize ear grains, 100-kernel weight, grain yield, and biomass yield from 2013 to 2016. Treatments N450, N300, N150, and N0 represent applied N:P_2_O_5_ rates of 0:0, 150:75, 300:150, and 450:225 kg hm^–1^, respectively.

There was significant different in grain yield between different year, which increased significantly (*P<0.05*) by an average of 59.70% under all fertilization treatments compared to the RN (**Figure 8**). However, there was little variation among the fertilization treatments. Except for the 2013, in which RL yielded higher than RH and RM, the order of fertilization treatments was RM > RH > RL in the other years. Only in 2014, the yield significantly (*P< 0.05*) increased under RM by 8.34% compared with RL, while there were no significant differences among the other treatments.

The biomass yield followed a similar trend to the grain yield, with all fertilization treatments showing significant increase (*P< 0.05*) compared to RN, by 51.28%. However, the differences between the fertilization treatments decreased gradually with years of fertilization. In 2013, RH significantly (*P< 0.05*) increased by 4.07% and 4.29% compared with RM and RL, respectively, and RM was 20.98% (*P< 0.05*) and 9.69% (*P< 0.05*) higher compared with RL. No significant differences were observes among other treatments in each years.

### 3.4 N and P utilization under different fertilizer rates

**Figure 9:**
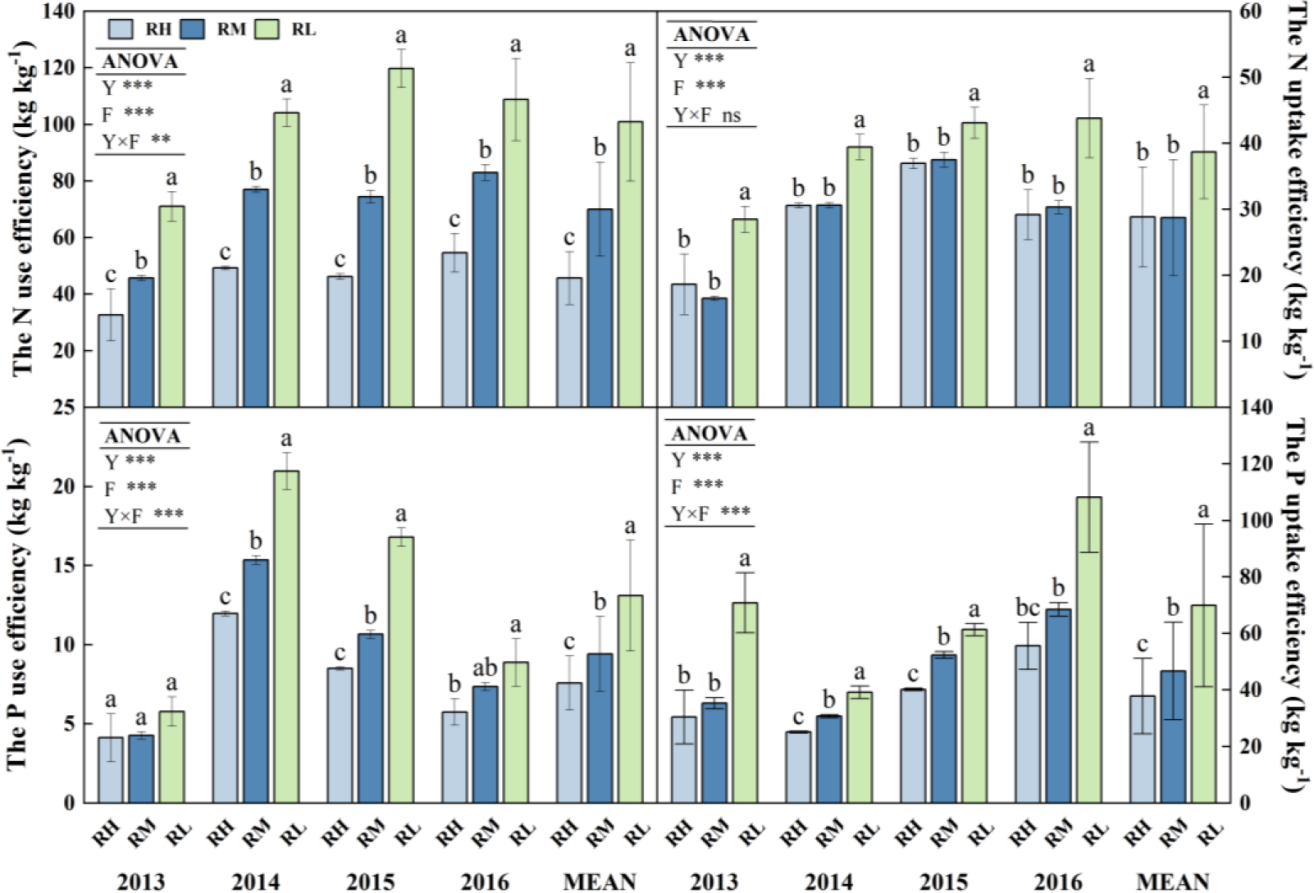
N and P use efficiency and uptake efficiency of maize under different fertilization levels from 2013 to 2016. Treatments RH, RM and RL represent applied N:P_2_O_5_ rates of 0:0, 150:75, 300:150, and 450:225 kg hm^–1^, respectively. Data are means ± SD (n=3). Lower case letters indicate significant differences among treatments. Bars represent standard deviations (LSD test, P< 0.05). In Analysis of Variance, Y, F and Y×F represents the year, fertilizer and the interaction between the year and fertilizer. *** Significant differences at P < 0.001; ** significant differences at P < 0.01; ns indicates non–significant difference.

**Figure 10:**
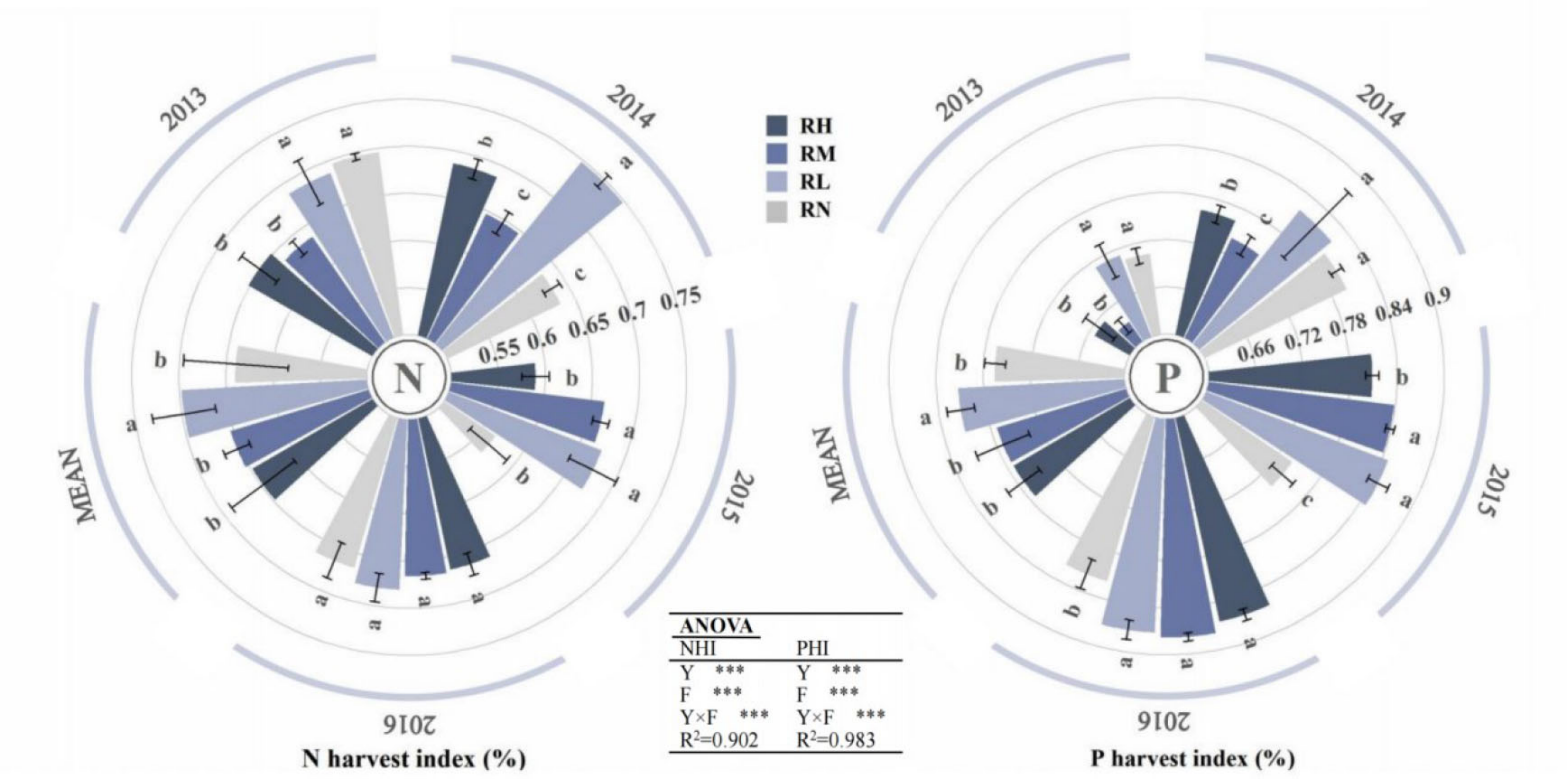
N and P harvest index of maize under different fertilization levels from 2013 to 2016. Treatments RH, RM, RL, and RN represent applied N:P_2_O_5_ rates of 0:0, 150:75, 300:150, and 450:225 kg hm^–1^, respectively. Data are means ± SD (n=3). Lower case letters indicate significant differences among treatments. Bars represent standard deviations (LSD test, P< 0.05). In Analysis of Variance, Y, F and Y×F represents the year, fertilizer and the interaction between the year and fertilizer. *** Significant differences at P < 0.001.

The N fertilizer use efficiency, uptake efficiency, and harvest index of maize were significantly (*P< 0.001*) affected by the year and fertilization rate in each year (**Figures 9**, **10**). The N fertilizer use efficiency of all treatments gradually decreased with fertilization rate increase, and the RL significantly (*P< 0.05*) increased by 54.47% and 30.80% compared to the RH and RM, respectively, whereas the RM increased by an average of 34.17% (*P< 0.05*) compared to the RH. In addition, the order of N fertilizer uptake efficiency was RL > RM > RH except for 2013 (RL > RH > RM), and the RL significantly (*P< 0.05*) increased by 22.05% and 23.39% compared to the RM and RH, respectively, but with no significant difference between RH and RM in 2014-2016. During the all experimental, the N fertilizer harvest index under RL was the highest, and the RL significantly (*P< 0.05*) increased by 6.71% and 10.51% compared to the RH and RM in 2013-2014. In 2015-2016, the order of N fertilizer harvest index of each fertilization treatments was RL > RM > RH, and the RL and RM increased by an average of 7.52% (*P< 0.05*) and 5.83%, respectively, compared to RH.

The year and fertilization rate on P fertilizer use efficiency, uptake efficiency, and harvest index of maize were extremely significant (*P< 0.001*). The order of P fertilizer use efficiency for all treatment was RL>RM>RH, and RL significantly increased (*P<0.05*) by 39.05% and 26.63% compared to RH and RM, respectively, RM showed an average increase of 16.91% (*P<0.05*) compared to RH. Moreover, the P fertilizer uptake efficiency of all treatments gradually increased with the fertilization years, and the order was RL>RM>RH. The P uptake efficiency of RL and RM was 43.99% (*P<0.05*) and 18.48% higher than RH, respectively. Furthermore, there were differences in the phosphorus harvest index of each treatments among different years. In 2013-2014, the order of each treatments was RL>RN>RH>RM, the RL significantly (*P<0.05*) increased by 7.56% (*P<0.05*), 10.38% (*P<0.05*), and 1.54% compared to RH, RM, and RN, respectively, and the RH was 3.05% higher compared to RM. In 2015-2016, all fertilizer treatments were significantly higher (*P<0.05*) than RN, with average increases of 6.67% and 12.46%, respectively. Among the fertilization treatments, only RL and RM significantly increased (*P<0.05*) by 4.87% and 3.73% in 2015 compared to RH, respectively, but there were no significant differences between other treatments.

## 4 Discussion

### 4.1 Growth and yield effects of fertilizer rate in RFRH system

**Figure 11:**
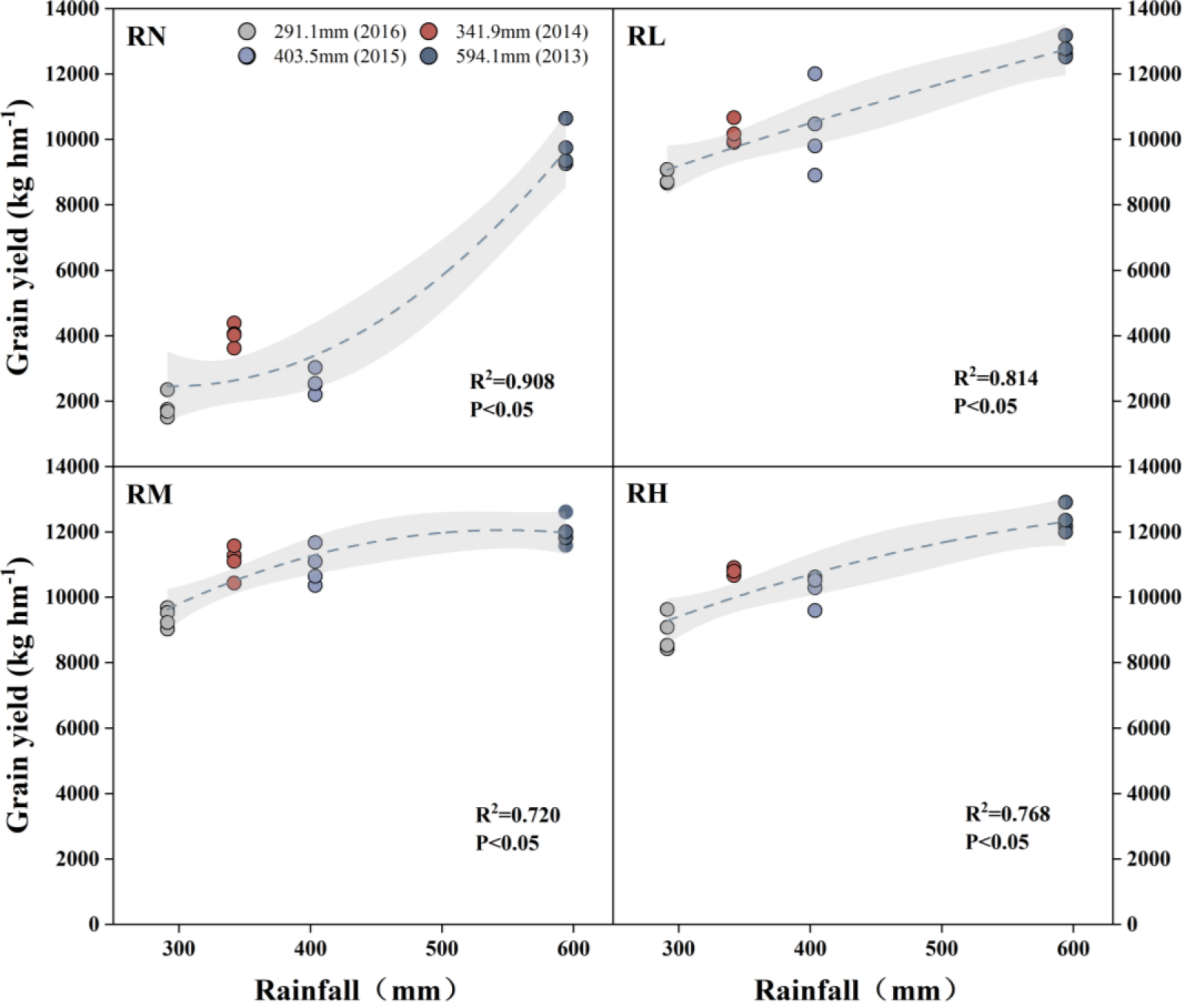
Linear regression relationship between maize grain yield and annual precipitation under different fertilization levels from 2013 to 2016. Treatments RH, RM, RL, and RN represent applied N:P_2_O_5_ rates of 0:0, 150:75, 300:150, and 450:225 kg hm^–1^, respectively. The dots in the graph represent annual precipitation for different years: 594.1mm (2013), 341.9mm (2014), 403.5mm (2015), 291.1mm (2016).

N and P are the essential nutrients for plant growth and development, and appropriate fertilization management has a positive impact on crop growth, yield formation, and ecological environment protection in farmland (Duan et al., 2011; Wu et al., 2018). In this study, the plant height and leaf area of maize were increased with fertilizer application rates increase during 2013-2014, this may be due to the abundant rainfall increased soil moisture and nutrient availability. Moreover, the cumulative effect of fertilizer decreased the maize plant height with high fertilization rates in 2016, whereas the height was highest under RL and leaf area was highest under RM in 2015-2016. The consecutive fertilizer application resulted in the gradual accumulation of N and P residues in the soil, and AE_NP_ (agronomic efficiency of fertilizer) was decreased with the increase of fertilizer rate (**Figure 11**). In addition, due to insufficient rainfall and dry conditions, the maize physiological growth processes was delayed, which could affect the crop water and fertilizer utilization, then weaken the water-fertilizer coupling effect even resulting in a negative effects (Lian et al., 2016; Li et al., 2018).

Under the RFRH system, the appropriation fertilizer application rate could coordinate the source-sink relationship of crops, and laying a foundation for high yield (Wu et al., 2022). Wei et al. (2019) suggested that the maize yield was determined by the sink capacity, which is affected by both ear grains and 100-kernel weight. This study indicate that fertilizer application had a significant effects on the dry matter accumulation and grain yield of maize, and the average value for four years was gradually increased with fertilizer rates increase (**Figure 5**). Because N and P fertilizers combined application could increase synthesis of active oxygen scavenging enzymes in crops, promote chlorophyll synthesis, transfer photosynthetic products to the grains, as well as increasing grain filling rates, thereby effectively improving maize sink capacity (ear grains) and filling degree (100-kernel weight) (Zhang X. D, et al., 2021; Fernandes et al., 2022; Nasar et al., 2022). Our study also showed that maize yield and dry matter accumulation generally increased and then decreased with fertilizer application rates increase, reaching a maximum value under RM, which is consistent with the results of Morell et al. (2011). The linear fitting also showed that grain yield, biomass, 100-kernel weight, and ear grains all followed a parabolic trend with increasing fertilizer application rates, reaching a peak value at RM, especially with the increase of fertilization years (**Figure 8**). This may be the results of both N and P fertilizer factors. Because the soil phosphorus was gradually accumulates with increasing years of fertilizer application, accelerating vegetative growth of crops, leading to premature development of reproductive organs, affecting the ear grains and grain weight of maize (Chen et al., 2015; Kong et al., 2017). Another, excessive N fertilizer application resulted in premature leaf senescence, reduced photosynthetic capacity, which affected the carbon assimilation process and decrease nitrogen proportion of maize grains, leading to the sterilization of maize grain (Liu et al., 2018; Loudari et al., 2022). Eventually, the combination of these two factors reduced maize yield.

### 4.2 Effects of nutrients uptake under fertilizer rates

The nutrients content and uptake in various plant organs were two important factors for assessing nutrient uptake and utilization in crops (Luo et al., 2016). In this study, fertilization treatments were significantly increased the accumulation of nitrogen and phosphorus, with an average increase by 260.53% and 264.74% compared to the RN, respectively. The above-ground biomass of maize significantly increased after fertilization under RFRH system. Because film mulching could effectively improve soil moisture and temperature conditions to promote the maize roots and canopy growth, thus increasing nutrient accumulation. Similar results was also obtained by Li et al. (2019) in the arid region of northwest China.

Li et al. (2021) found that crop plants nitrogen accumulation was initially increased and then followed by a decrease with fertilizer application rates. Similarly, our study also found that the increase in nitrogen accumulation was highest in RM treatments (**Table 1**). Because appropriate nitrogen application rate can effectively promote crop nitrogen accumulation, while excessive rate could alter soil structure, acidity, as well as soil moisture and temperature conditions, thereby affecting the survival environment of soil microorganisms and roots growth, resulting in negative effects and reduced nutrient uptake (Zhang et al., 2016; Wu et al., 2020). In contrast to the results of Zhang Y. M, et al. (2021) on maize phosphorus uptake in the Loess Plateau region, our results showed an upward trend in phosphorus accumulation with fertilizer application rates increase (**Table 1**). This may be related to different planting areas and soil nutrient contents, or it may be due to nitrogen application promotes aboveground growth and significant increases biomass yield. Another possibility was that the enzymes involved in nitrogen metabolism could also promote phosphorus absorption, thereby increasing the amount of phosphorus uptake, which did not reach the critical phosphorus uptake threshold (Jing et al., 2022). Additionally, differences between years was attributed to the influence of climates in different experimental years, and the N and P accumulation of maize during 2013-2014 was significantly higher than that during 2015-2016. This was likely due to abundant rainfall and favorable rainfall distribution for maize growth in 2013 and 2014, which could provide an ideal water-fertilizer coupling condition for crop growth, thus stimulating the growth-promoting effects on maize. Moreover, it also promoted the transformation of soil nitrate nitrogen and available phosphorus, thereby enhancing maize nutrient uptake (Dong et al., 2019).

The coordinated supply of nitrogen and phosphorus could promote the nutrient absorption and utilization of crop, and was also beneficial to the growth and yield increase (Phares et al., 2022). This study found that the trends of nitrogen and phosphorus accumulation in different parts of maize plants in different fertilizer rates treatments was different in different experimental years (**Figure 7**). It indicates that the nutrient transfer from vegetative organs to reproductive organs under different precipitation patterns were different. Moreover, our results also showed that there was obvious threshold effect in fertilization promoting nutrient accumulation and yield increase. Because soil water participates in many growth processes, such as synthesis of biological macromolecules and enzymes, plant physiology and biochemical, thus the nutrient uptake and utilization depend on soil moisture effectiveness. Hu et al. (2009) found that soil moisture content determines the availability of soil nitrogen and its transport to the roots. Under dry conditions of the Loess Plateau, water stress limits both nitrogen mineralization and root nutrient uptake, thereby weakening the crop yield response to nitrogen input (Lian et al., 2017). The application of phosphorus fertilizer can promote rapid root development and branching, indirectly improving nitrogen uptake. When only phosphorus fertilizer is applied, it promotes the growth of a small amount of roots, whereas the impact of single nitrogen fertilizer application is relatively small, and it even increases the risk of nitrogen leaching (Wen et al., 2016). In addition, when sufficient water is available, leaf photosynthesis and root activity are enhanced, achieving more effective transportation and accumulation of nutrients (Qi et al., 2020). Alternation of wet and dry conditions can also change soil structure, affecting the adsorption and release of available nutrients by soil aggregates (Xiang et al., 2008).

### 4.3 Effect of N and P utilization under fertilizer rates

The RFRH systems can increase crop yield and fertilizer utilization efficiency. However, the utilization efficiency generally shows a downward trend as fertilizer application increased, and the highest yield does not always correspond to the highest utilization efficiency (Zhang et al., 2015; Wang et al., 2018). Similarly, in this study, compared with RL, the RM achieved higher N/P accumulation and maize yield, but with significantly lower nitrogen and phosphorus use efficiency, uptake efficiency, and harvest index. While the nitrogen and phosphorus fertilizer use efficiency, uptake efficiency, and grain yield of RM were moderately enhanced than RH. This study showed that the nitrogen use efficiency, uptake efficiency, and harvest index of maize decreased gradually with fertilizer application rates increase, and reaching the maximum value under RL (**Figures 9**, **10**). This is consistent with the results of a long-term located field experiment by Yang et al. (2017). It may be due to the fact that the nitrogen fertilizer exists in the form of nitrate nitrogen in alkaline soils, and when the fertilizer application rate is too high and the inorganic nitrogen content exceeds the safe threshold, excessive nitrogen was leached, thus reduced nitrogen fertilizer use efficiency (Ju et al., 2004; Woli et al., 2016). Similarly, this study also showed that the phosphorus fertilizer use efficiency and uptake efficiency of maize decreased with fertilizer application rate increase, and the phosphorus fertilizer use efficiency was gradually decreased with the increasing of fertilizer application years (**Figures 9**, **10**). This may be related to the fact that phosphorus was a fast-acting fertilizer, and it was easily strongly adsorbed and further mineralized by the soil (Yuan et al., 2022). Continuous fertilizer application leads to an increasing amount of unused phosphorus in the soil, which hinders its absorption and utilization. Moreover, the nitrogen and phosphorus use efficiency of each fertilization treatment have differences with the fertilizer application rate under different precipitation years. When rainfall is abundant (2013), it may have further exacerbated nitrogen and phosphorus nutrient leaching, while reducing soil cation exchange capacity. Meanwhile, excessive application of inorganic fertilizers also exacerbates soil nitrification, causing soil acidification and the soil phosphorus content may have been relatively low in the early stages of the experiment, resulting in lower nitrogen and phosphorus utilization efficiency in 2013 (Duan et al., 2014; Guo et al., 2017). The appropriate alternation of dry and wet seasons (2014) promoted the release of phosphorus in soil and increased its solubility, greatly improving the effectiveness of phosphorus in soil (Bünemann et al., 2013; Suriyagoda et al., 2014). And good water-fertilizer effects resulted in high N and P fertilizer use efficiency with moderate rainfall in 2015. When rainfall is insufficient in 2016, the evapotranspiration was increased with fertilizer application, thereby exacerbating water stress, reducing water use efficiency and restricting root and rhizosphere growth, which was not conducive to the early growth and development, affects nutrient accumulation, and leads to a decrease in nitrogen use efficiency (Li et al., 2018; Zhang et al., 2022).

## 5 Conclusion

The RFRH system can effectively promote the water- fertilizer relationship of crops. The results of this study indicate that under sufficient precipitation, the RM treatment obtained the highest yield, while the RL treatment had the highest nitrogen and phosphorus utilization efficiency. Under less precipitation, the water-fertilizer relationship was somewhat inhibited and affected the yield, but the RM treatment still had highest dry matter, yield and nutrient accumulation, while the fertilizer utilization efficiency was little lower than the RL. Overall, the medium fertilizer rate (N 300 kg hm^-2^, P_2_O_5_ 150 kg hm^-2^) was recommended as the suitable fertilizer rate for RFRH system in semi-arid regions, additionally, reduce the fertilization appropriately based on the precipitation amount.

## Data availability statement

The original contributions presented in the study are included in the article/supplementary material. Further inquiries can be directed to the corresponding author.

## Author contributions

The manuscript was reviewed and approved for publication by all authors. XR, ZJ and PZ conceived and designed the experiments. JW, GL, NC, YZ, and DL performed the experiments. JW, GL, NC, YZ, and DL analyzed the data. JW, YZ, and DL wrote the paper. EL, PZ, XR, and ZJ reviewed and revised the paper. PZ and EL corrected the English language for the paper.
